# The Endoscopic Endonasal Dacryocystorhinostomy (eDCR) in the Immunocompromised Patient

**DOI:** 10.1007/s12070-023-03515-9

**Published:** 2023-02-05

**Authors:** G. Lunardi, P. Schiavo, R. Amadori, M. Cellina, G. Termine

**Affiliations:** 1 Department of Otolaryngology, Ospedale Fatebenefratelli E Oftalmico, Università Degli Studi Di Milano, Piazza Principessa Clotilde 3, 20121 Milan, Italy; 2 Department of Otolaryngology, Azienda Socio Sanitaria Territoriale Fatebenefratelli Sacco, Ospedale Fatebenefratelli E Oftalmico, Milan, Italy; 3 Department of Obstetrics and Gynecology, Azienda Ospedaliera Universitaria Maggiore Della Carità, Novara, Italy; 4 Department of Radiodiagnostics, Azienda Socio Sanitaria Territoriale Fatebenefratelli Sacco, Ospedale Fatebenefratelli E Oftalmico, Milan, Italy

**Keywords:** Sinus surgery, Endoscopic surgery, Eye, Orbit, Immunology

## Abstract

We present the clinical case of a 51-year-old male patient, affected by common variable immunodeficiency (CVID). In his history recurrent orbital cellulitis, exacerbation of chronic right dacryocystitis, lacrimal sac empyema with periodic episodes of dacryocutaneous fistolization. The coexistence of these particular immunological defects and the lack of literature about similar cases required an accurate evaluation of each step of the diagnostic and therapeutic approach. We performed an endoscopic endonasal dacryocystorhinostomy with “cold” instruments. No surgical complications were observed in the immediate postsurgical period. We balanced the necessity of a follow-up based on frequent office evaluation and the current pandemic emergency, in order to not expose the patient to an additional infectious risk. The discussion will focus on several aspects: the adequacy of radiological, the “cold” surgical technique, the choice of avoiding endocanalicular prostheses. We will discuss also about the use of oral and topical therapy, avoiding probable post-surgical infectious complications.

## Introduction

The endoscopic endonasal dacryocystorhinostomy (eDCR) is a surgical technique designed in the late 1800s to solve a stenosis of the distal lacrimal duct, avoiding possible serious complications caused by bacterial superinfections.

The immunecompromission of our patient and the lack of literature about similar cases required an accurate evaluation of each step of the diagnostic and therapeutic approach.

In addition, the ongoing state of health emergency for COVID-19, which still complicates access to specialist care, played a main role in the management of this patient, in order to balance the necessity of a follow-up based on frequent nasal evaluations and medications, preserving him from other infections.

This is a particular case which could provide some basis also for the management of elderly patients in which the defensive capacity of the immune system is notoriously reduced, or for other individuals suffering from various forms of immunodepression.

## Case Report

We present the case of a 51-year-old male patient who came to our attention on the 19th of August 2020.

His medical history was significant for recurrent orbital cellulitis of the right eye with exacerbation of chronic dacryocystitis, lacrimal sac empyema and episodes of dacryocutaneous fistolization since 4 months.

The clinical management of this patient was complicated: he was allergic to amoxicillin and he was affected by common variable immunodeficiency (CVID), with low levels of IgM and IgGs.

**Fig. 1:**
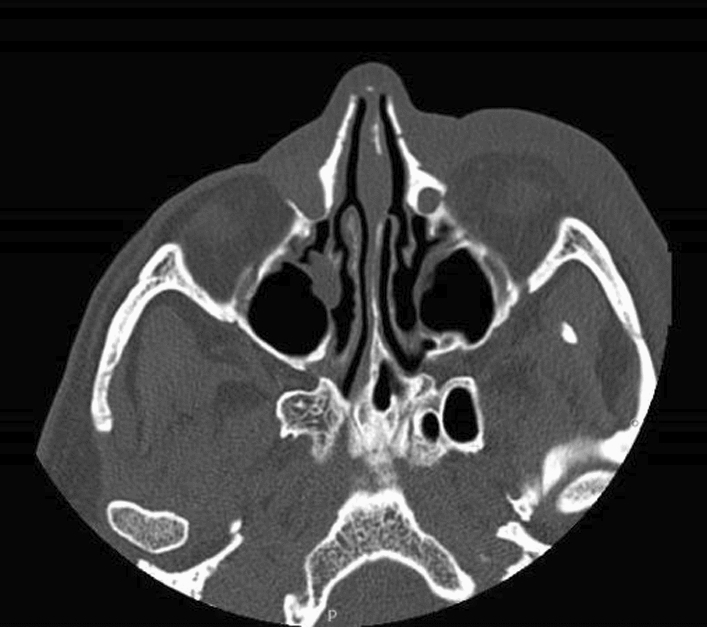
TC of the facial head without contrast This picture shows the anatomy of the facial massif of the patient and the dilation of the right lacrimal sac

The patient went to the Emergency Room (E.R.) at Fatebenefratelli Hospital in Milan, presenting with orbital cellulitis. In the E.R. a CT of the head without contrast was performed (Fig. 1) and stagnation of purulent material in the right lacrimal sac was documented. The patient was treated with clarithromycin 500 mg twice daily for 21 days (due to his amoxicillin allergy), nasal saline irrigation and corticosteroid nasal spray.

An ENT evaluation was required for possible right eDCR.

**Fig. 2:**
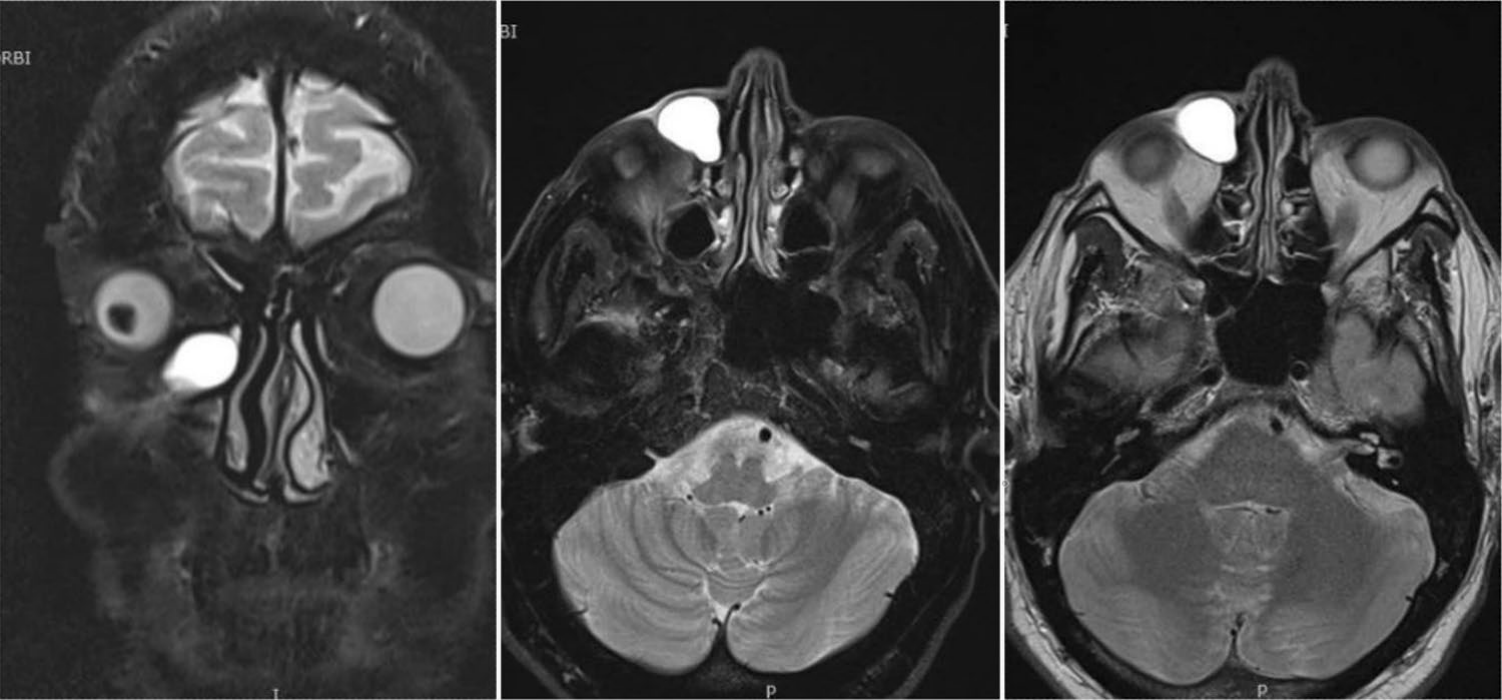
DacryoMRI of the head, T2-weighted With the help of an expert radiologist, the boundaries and dimensions of the lacrimal sac can be clearly highlighted, using simple artificial tear drops applied to the patient’s corneal surface and drained as a more physiological contrast

The patient underwent Dacryo-MRI [1], focused on axial and coronal acquisition of images, performed with T2-weighted images for enhancement of static fluids, STIR, T1 fat-sat (Fig. 2).

MRI displayed dilation of the right lacrimal sac, with purulent-appearance content (20 × 16 mm). No obvious changes in signal intensity at the retrobulbar fat tissue was noticed. Fortunately the exam showed no signs of rhinosinusitis, which would have required a clearance of the paranasal sinuses during the operation with lengthening of the surgical times and therefore an increase in the risk of surgical infections [2]

The nasal endoscopy with rigid optic completed the pre-operative planning, documenting a normal nasosinusal anatomy.

The patient underwent surgery on the 13th of November because the logistic aspect in relation to the concomitant state of health emergency for COVID-19 complicated the access to elective surgery in our hospital (COVID-19 Hub Hospital). So we recommended daily nasal washes and re-evaluated the patient every 15 days to detect early symptoms of infection.

Then we performed an eDCR with “cold” instruments.

No complications were observed in the early postoperative time.

At home, antibiotic therapy was set up: oral Cefditoren 400 mg every 12 h for 10 days and Tobramycin eye drops, along with nasal irrigations with isotonic solution, nasal emollient therapy, a painkiller as needed, and prescription of massage of the lacrimal sac three times a day.

**Fig. 3:**
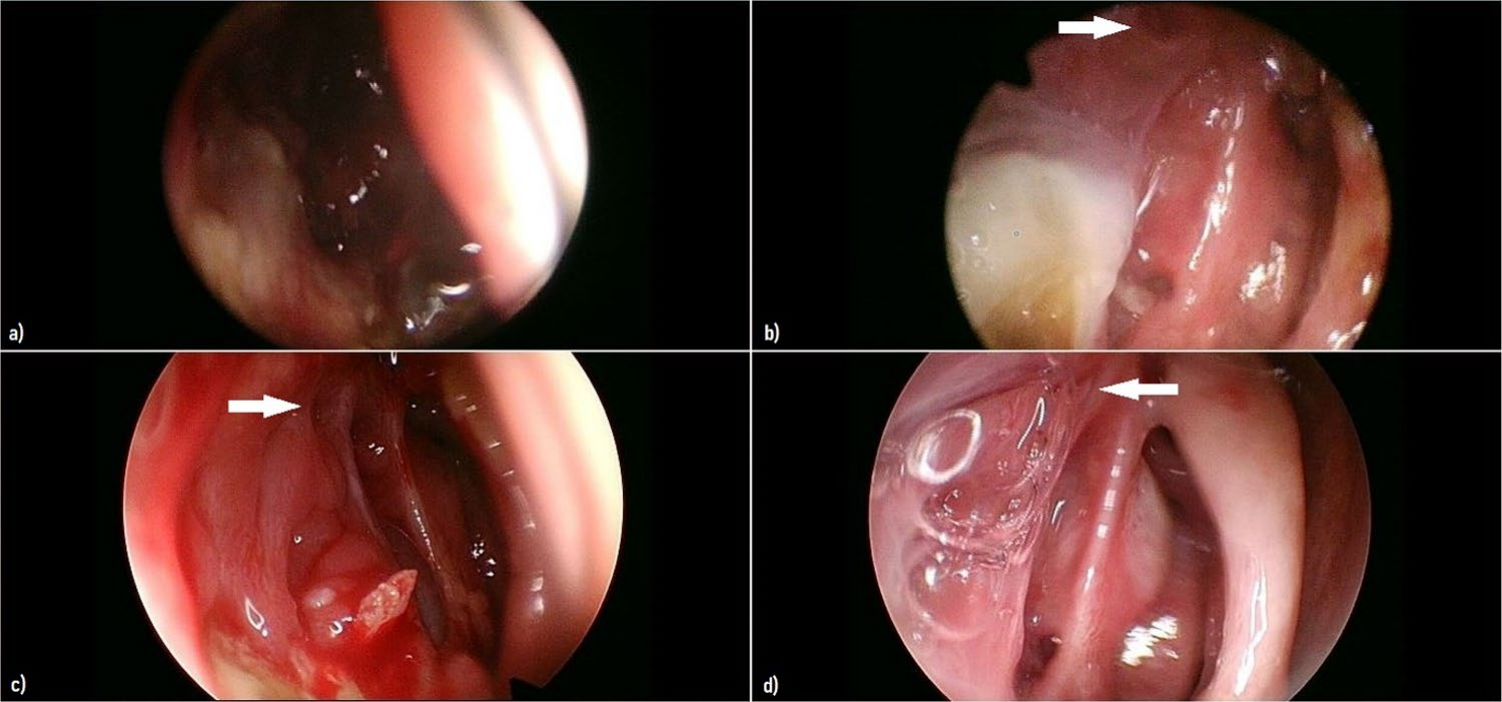
Images from nasal endoscopies of the follow-ups White arrows indicate the site of the dacryocystorhinostomy. **a** 1 week postoperative follow-up, **b** 3 weeks p.o. follow-up, **c** 1 month p.o. follow-up, **d** 2 months p.o. follow-up

To monitor the surgical results, the patient underwent periodic nasal endoscopies with the Olympus Evis Exera III CV-190 with 4 K resolution images (Fig. 3).

During the first post-operative month the clinical development was regular, with slight stagnation of purulent secretions, predictable due to the underlying immunodepression, without pathological significance.

At first and second months’ follow-ups the surgical result appeared excellent, with well-defined edges of the dacryocystorhinostomy.

**Fig. 4:**
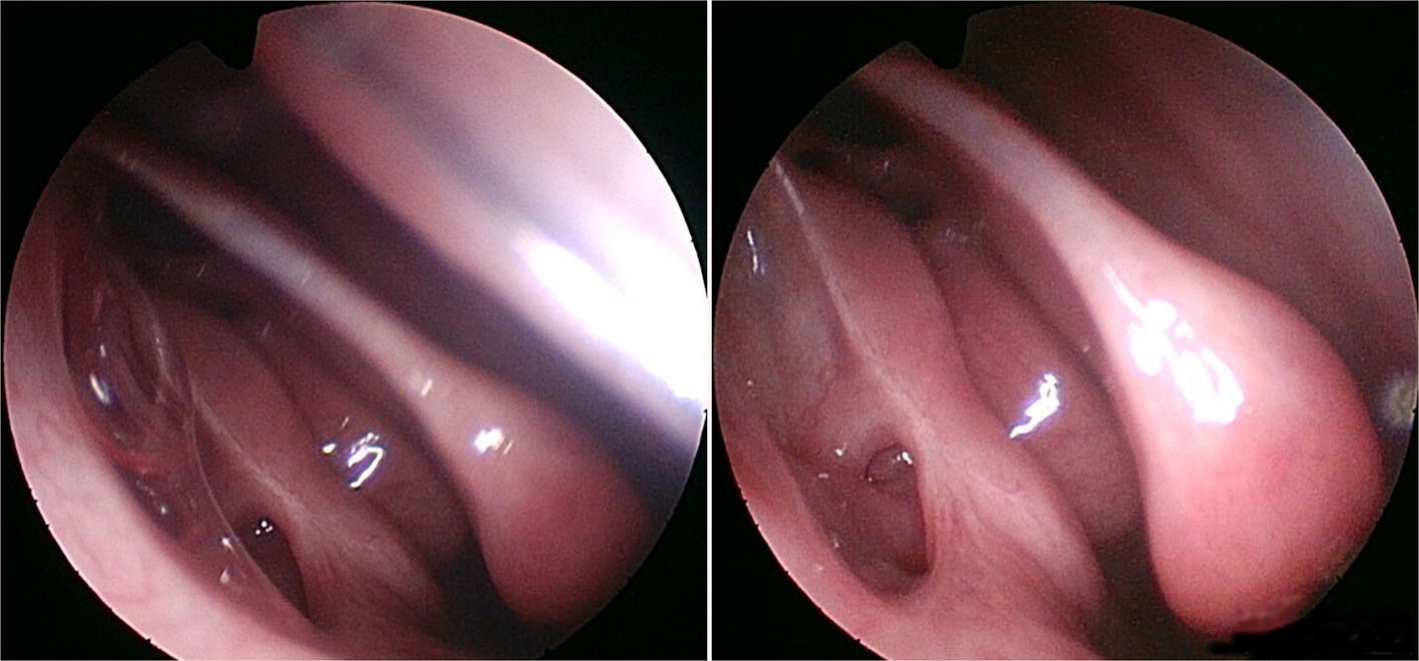
Final result (six-months follow-up)

The nasal evaluation at the sixth post-surgical month (Fig. 4) confirmed spontaneous tear drainage as in the previous checks.

The patient is currently continuing only with daily nasal washes and artificial tears as needed.

## Discussion

We want to focus on several aspects: the use of a “cold” surgical technique; our choice to avoid endocanalicular prostheses; the use of balanced oral and topical therapy.

Despite the lack of decision-supporting literature and the rate of restenosis that is still around 10–15%, the sixth-months result seems to confirm the effectiveness, stability and safety of the use of pure-cold technique in an immunocompromised patient.

Although surgical technique doesn’t seem to directly affect the formation of postoperative infections, we opted for a “cold” surgery due to the lower rate of formation of crustiness and synechiae that indirectly would have promoted stagnation of secretions and could have lead to bacterial proliferation. Standard endoscopic DCR and its more sophisticated modifications are equally effective and safe in managing distal nasolacrimal drainage obstruction [3] and in our experience a standard endoscopic DCR performed by an expert surgeon, is as fast as other techniques.

We underline our particular care in avoiding the use of hot tools to control intraoperative bleeding, preferring topical decongestion with nasal sponges medicated with vasoconstrictor (epinephrin diluted 1:100,000) [4].

Moreover the mucoperiosteal flap, the lacrimal osteotomy and the dacryocystorhinostomy were all performed with cold techniques, no drilling was used.

We decided not to place silicone tear stents to ensure the patency of the tear pathway because they represent a substrate for bacterial colonization with biofilm formation [5].

In fact we found only one study in NCBI and in Cochrane Library databases, carried out on patients without comorbidity, where bacterial colonization does not seem to imply future clinically-relevant infections [6], and we did not make the patient run the risk [7].

Right nasal cavity was medicated with haemostatic, not-absorbable nasal sponge soaked with antibiotic oinment (Chlortetracycline) and tranexamic acid.

To increase our degree of safety in this patient we could have used resorbable sponge, which isn’t site of postoperative colonization, if it was been available in our operating room.

Focusing on the choice of antibiotic, we considered a study regarding bacterial coltures on stents removed after surgery [8]. The proximal portion of the stent is a site of colonization in one-third of patients without comorbidity; this colonization occurs mainly against Gram-negative bacteria. This topic lead us to the choice of a third generation Cephalosporin associated with eye drops based on Tobramycin (that intercepts the Gram-negative of the proximal portion of the lacrimal pathway).

Moreover, since one-third of the colonizations were made by the same microorganisms as the distal part of the stent and therefore probably climbed back from the nasal cavities [8], we were further convinced not to place the stents.

In conclusion, in order to summarise the key-points in the management of this type of patient, we believe that careful preoperative assessment is essential for a targeted and minimally traumatic approach, giving priority, if possible, to the use of cold tools.

We don’t recommend the use of endocanalicular prostheses due to the excellent results obtained, but we aren’t sure that positioning the stents the results would have been different.

We suggest to closely follow-up the patient, not underestimating the role of adjuvant medical therapy and not forgetting to control the underlying pathology that is the source of almost all the problems of these patients.
